# Detection of mutations in the *rpoB* gene of rifampicin-resistant *Mycobacterium tuberculosis* strains inhibiting wild type probe hybridization in the MTBDR plus assay by DNA sequencing directly from clinical specimens

**DOI:** 10.1186/s12866-020-01967-5

**Published:** 2020-09-16

**Authors:** Pallavi Sinha, G. N. Srivastava, Rajneesh Tripathi, Mukti Nath Mishra, Shampa Anupurba

**Affiliations:** 1 Department of Microbiology, Institute of Medical Sciences, Banaras Hindu University, Varanasi, 221 005 India; 2 Department of Respiratory Diseases, Institute of Medical Sciences, Banaras Hindu University, Varanasi, Uttar Pradesh 221005 India; 3 CSIR-Central Institute of Medicinal and Aromatic Plants, Lucknow, 226015 India

**Keywords:** DNA sequencing, Genotype MTBDR*plus*, Line probe assays, *Mycobacterium tuberculosis*, Reference standard

## Abstract

**Background:**

The potential of genetic testing for rapid and accurate diagnosis of drug-resistant *Mycobacterium tuberculosis* strains is vital for efficient treatment and reduction in dissemination. MTBDR plus assays rapidly detect mutations related to drug resistance and wild type sequences allied with susceptibility. Although these methods are promising, the examination of molecular level performance is essential for improved assay result interpretation and continued diagnostic development. Therefore this study aimed to determine novel mutations that were inhibiting wild type probe hybridization in the Line probe assay by DNA sequencing. Using data collected from Line Probe assay (GenoType MTBDR*plus* assay) the contribution of absent wild type probe hybridization to the detection of rifampicin resistance was assessed via comparison to a reference standard method i.e. DNA sequencing.

**Results:**

Sequence analysis of the *rpoB* gene of 47 MTB resistant strains from clinical specimens showed that 37 had a single mutation, 9 had double mutations and one had triple mutations in the *ropB* gene.

**Conclusions:**

The absence of wild type probe hybridization without mutation probe hybridization was mainly the result of the failure of mutation probe hybridization and the result of the novel or rare mutations. Additional probes are necessary to be included in the Line probe assay to improve the detection of rifampicin-resistant *Mycobacterium tuberculosis* strains.

## Background

Tuberculosis (TB), caused by *Mycobacterium tuberculosis* complex (MTBC), one of the world’s life-threatening communicable diseases and it ranks alongside the human immunodeficiency virus (HIV) as a leading cause of death [1]. The current increase in drug-resistant strains due to unsuccessful treatment and increased dissemination of resistance exhibits an important problem for global TB control efforts [2]. The diagnosis of tuberculosis depends on strong laboratory examination and skilled and dedicated personnel for treatment inaccuracy and supervision.

Conventional culture-based drug susceptibility testing methods take 4–8 weeks to yields results [3]. During this time, patients may be taking chemotherapeutics that are completely ineffective, and risk instantly transmitting the resistant disease to other persons. Therefore, there is an imperative need for rapid diagnosis of drug-resistant TB strains.

The WHO has endorsed the commercially available Line Probe Assay (LPA), the GenoType MTBDR*plus* assay (Hain Lifescience, Nehren, Germany) [4, 5]. The GenoType MTBDR*plus* assay detects the presence of *M. tuberculosis* together with the most common genetic mutations that conferring resistance to rifampicin (RIF) (mutations within the *rpoB* gene) and isoniazid (INH) (mutations within the *katG* gene and the *inhA* promoter), [6–8] and its diagnostic presentation has been evaluated in smear-positive pulmonary samples [6]. The diagnosis by the MTBDR*plus* assay depends on the amplification of gene regions known to bear resistance-associated mutations, as intent by reverse hybridization to wild-type (wt) and mutated sequences.

Rifampicin is one of the most potent first-line anti-TB drug that serves as a surrogate marker for the detection of multidrug resistant-tuberculosis (MDR-TB), as > 90% of Rif^r^ isolates are also isoniazid-resistant, another potent first-line anti-TB drug [9–11].

MTBDR*plus* assay has certain limitations in that MTB strains could have uncommon mutations. Few studies have reported that MTB strains have uncommon mutations in the *rpoB* gene [12–14]. Thus, it is essential to determine the mutation patterns among a large number of MTB isolates from various parts of India, since this would help not only in the design of a desirable diagnostic method for rapid detection of MDR-TB but also in the identification of any hot-spot regions in the different countries for the proper execution of TB control programs.

The present study aimed to determine novel mutations those were inhibiting WT probe hybridization in the Line Probe assay (Genotype MTBDR*plus* assay) by DNA sequencing.

## Results

A total of 150 AFB smear-positive pulmonary samples were analyzed using the GenoType MTBDR*plus* assay. Out of 150 samples, in 35 samples MTB was not detected, 98 samples were RIF resistant, and 17 samples identified as susceptible to RIF. Using the GenoType MTBDR*plus* assay, corresponding resistance rates were 65.3% (98/150). The most common genetic mutation conferring RIF resistance was Ser531Leu of the *rpoB* gene, detected in 63.3% (62/98) of RIF resistant strains. The next most frequent *rpoB* mutation encountered was Asp516Val which was detected in 21.4% (21/98) RIF resistant strains, followed by His526Tyr 12.2% (12/98) and combined His526Tyr, Ser531Leu in 3.1% (3/98) of the tested specimens.

Thirty-one percent (47/150) RIF resistant strains detected from clinical samples had non- interpretable (NI) results that could not be identified by GenoType MTBDR*plus* assay, missing.

bands on the wild type region of these strains but no hybridization with mutation probes suggested resistance due to mutations other than those included in MTBDR*plus* assay.

### Indeterminate GenoType MTBDRplus assay results for RIF resistant clinical specimens

**Fig. 1:**
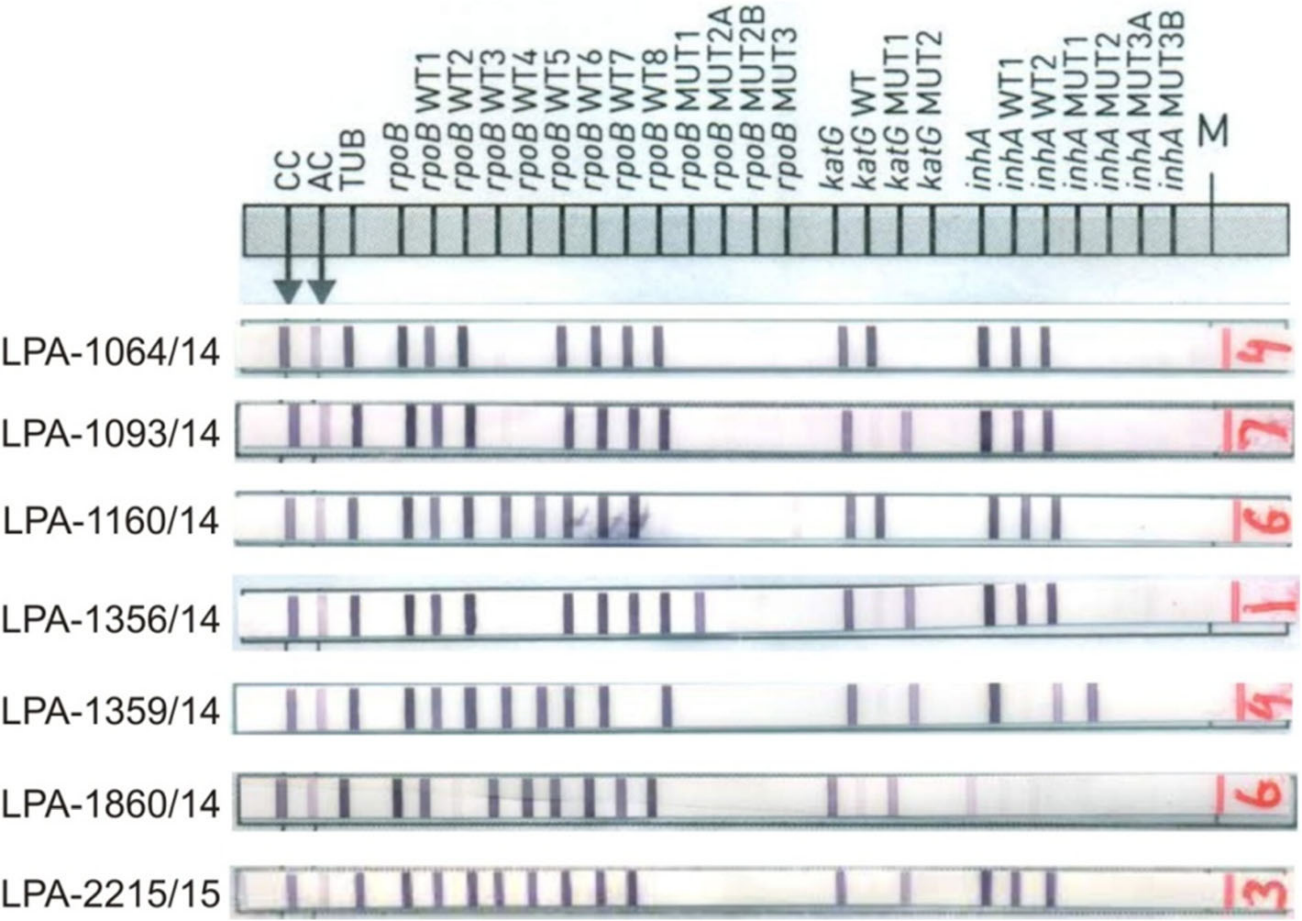
Strips of hybridization patterns of rifampicin resistance in MTBDRplus assay

Indeterminate results come about alongside for RIF in 31.3% (47/115) of the tests. The patterns of non-interpretable mutations associated with rifampicin resistance MTB strains in 47 samples are shown in Table 1. In 47 RIF resistant strains, one or more wild type probes were missing with no bands in mutant probes (Fig. 1). These 47 strains included missing WT8 (14; 29.8%), missing WT3&WT4 (16; 34%), missing WT2 (4; 8.5%), missing WT7 (5; 10.6%), missing WT3 (3; 6.4%), missing WT1,WT3&W4 (1; 2.1%) missing WT5 (1; 2.1%), missing WT4 (2; 4.2%) and missing WT5&WT6 (1; 2.1%). DNA sequence analysis of the 47 RIF resistant MTB strains from clinical specimens showed that 37 had a single mutation, 9 had double mutations and one had triple mutations in the *rpoB* gene.

**Table 1:** Pattern of gene mutations in *rpoB* gene detected by Genotype MTBDR*plus* and DNA sequencing of the *rpoB* gene for pattern of resistance in MTB strains

			Resistance classification		
Gene	Codons	Number by drug resistance category and ***rpoB*** gene loci	Gene region	Absent wild type probe	DNA sequencing results
*rpoB*	**531 (14)**	9, 1, 1, 1	531–533	WT8^a^	TCG-TGG (Ser-Trp), TCG-TGC (Ser-Cys), TCG-TAT (Ser-Tyr), TCG-TTT (Ser-Phe)
	531&526	1	531–533	WT8^a^	TCG-TGG (Ser-Trp) & CAC-TAC (His-Tyr)
	531&605	1	–	–	**TCG-TTC (Ser-Phe)** & TAC-GAC (Tyr-Asp)
	**526 (5)**	1, 1	525–530	WT7	**CAC-TGC (His-Cys)**, CAC-AAC (His-Asp)
	526, 506&531	1	525–530	–	**CAC-CTC (His-Leu)**, TTC-TTA (Phe-Leu) & TCG-TGG (Ser-Trp)
	526&528	1	–	WT7^a^	**CAC-CTC (His-Leu)** & CGC-TGC (Arg-Cys)
	526&506	1	–	WT7	**CAC-CTC (His-Leu)** & TTC-TTA (Phe-Leu)
	**516 (12)**	1	513–517	WT3^a^	Ins CTT (Leu) b/w 514–515
	**–**	1		WT3	GAC-TAC (Asp-Tyr)
	**–**	3, 6	513–517 & 516–519	WT3&4	GAC-GTC (Asp-Val), GAC-TAC (Asp-Tyr)
	509&516	1	506–509, 513–517 & 516–519	WT 1,3&4	AGC-ATC (Glu-His), GAC-TAC (Asp-Tyr)
	516	1	–	WT4^a^	GAC-GTC (Asp-Val)
	**513 (4)**	2,1,2		WT3&4	CAA-CCA (Gln-Pro), CAA-GAA (Gln-Glu), **CAA-GTA (Gln-Val)**
	513&526	1, 1	–	WT3&4	CAA-CTA (Gln-Leu) & CAC-CAG (His-Gln)
		1	–	WT3&4	Ins CTT (Leu) at 513–514
	**515&572**	1	513–517	WT3^a^	**ATG-CTG (Met-Leu)** & ATC-TTC (Ile-Phe)
	**511 (3)**	1, 2, 1	510–513	WT2	CTG-CCG (Leu-Pro), **Ins CCG (Pro) at 511–513**, **Ins GAG (Glu) at 511–512**
	**518 (3)**	2	518–522	WT5	AAC-GAC (Asn-Asp)
	**–**	1	518–522 & 522–525	WT5&6	Del AAC (Thr)
	518	1	516–520	WT4	AAC-GAC (Asn-Asp)

^a^indeterminate results

*Bold columns showing new mutations*

**Fig. 2:**
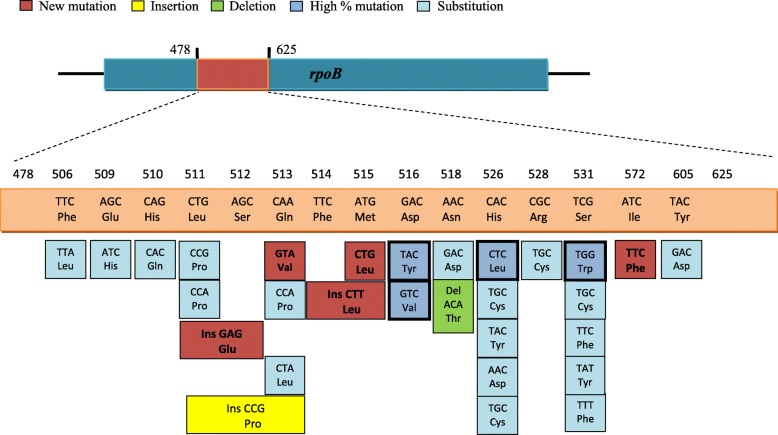
Depiction of rare mutations observed in the *rpoB* gene of RIF resistant *M. tuberculosis* strains from Clinical specimens

Fifteen different types of missense mutations were identified in 47 rifampicin-resistant strains from those with non-interpretable results at codons 516, 506, 509, 510, 511, 513, 515, 518, 526, 528, 531, 533, 572 and 605. Fifty different missense mutations were caused by single base substitutions (*n* = 26), deletions (*n* = 1) and insertions (*n* = 3; Table 1& Fig. 2). Fifteen strains had rare mutations at codon 531 (TCG-TGG, TTC, TGC, TAT, and TTT). Twelve strains had rare mutations at codon 516 (GAC-TAC, GTC, and TAC) and one strain contained an insertion of CTT between codons 514 and 515. Seven strains had rare mutations at codon 526 (CAC-TAC, CAG, AAC, CTC, and TGC). Eight strains had rare mutations at codon 513 (CAA-CCA, GAA, GTA, and CTA). One strain contained mutation at codon 515 (ATG-CTG), one strain contained mutation at codon 511 (CTG-CCG), two strains had insertion of CCG between codons 511 and 513 and one strain had insertion of GAG between codons 511 and 512. Two strains had mutations at codon 518 (AAC-GAC) and one strain had a deletion of ATA (Fig. 2).

Two strains contained mutation from TTC to TTA at codon 506, one strain contained mutation from CGC to TGC at codon 528, one strain had a mutation from CAC to CAG at codon 510, one strain had a mutation from AGC to ATC and one strain had a mutation at codon 533 from CTG-CCG (Table 1). Multiple sequence alignment result of 22 MTB strains that harbor most common and rare mutations in the *rpoB* gene have shown in supplementary file-1.

Two new mutations (TAC to GAC at codon 605 and ATC to TTC at codon 572) outside the 81-bp hot-spot region was also seen in two strains together double or triple mutations at different codons (Fig. 4). Ten new mutations were also recognized in this study. A mutation from TCG (Ser) to TTC (Phe) at codon 531, a mutation from CAC (His) to TGC (Cys) and a mutation from CAC (His) to CTC (Leu) at codon 526, a mutation at codon 513 from CAA (Gln) to GTA (Val), a mutation at codon 515 from ATG (Met) to CTG (Leu), insertion of CCG (Pro) between codon 511 and 513 and one insertion of GAG (Glu) between codon 511 and 512 (Table 1).

## Discussion

Rapid detection of genes associated with rifampicin-resistant in *M. tuberculosis* strains is a key challenge for the treatment of tuberculosis. Besides, during this time geographical observation of rising MTB drug resistance would make possible more appropriate antibiotic treatment approaches. At present, the GenoType® MTBDR*plus,* molecular methods offer limited detection, mainly when a novel or rare amino acid substitutions are inside known drug resistance regions. Genotypic test like GenoType® MTBDR*plus* assay has a disadvantage of any possibility of silent mutation, i.e. mutations which do not lead to change in amino acid hence not extending to phenotypic drug resistance. Therefore we have used the DNA sequencing method for the detection of rare or uncommon mutations. In addition, we don’t know if the new mutations detected are associated or not with RIF-resistance as drug susceptibility testing was not performed in this study.

Various studies have shown that resistance to INH is very common in high-TB-burden countries, and the isolate may not be resistant to RIF [15]. Conversely, if the isolate is RIF resistant, it is more likely that it is also INH resistant, thus making RIF resistance a surrogate marker for the identification of MDR-TB [16].

Seifert et al have studied that, for any gene region, the absence of the WT probe hybridization most often indicated a failure of MUT probe hybridization, rather than the presence of novel or rare mutations [17]. But in this study molecular analysis of the *rpoB* gene of *M. tuberculosis* clinical strains was carried out and rare and new mutations were identified by the DNA sequencing method. Some rare mutations at codon 531 (Ser to Trp/Cys/Phe/Tyr) were found in the *rpoB* gene of 32% strains through sequencing of RIF-resistant strains which is a rare mutation [18, 19]. Fifteen percent of the strains had the mutation at codon 526 (CTC, TAC, TGC, AAC) that resulted in the conversion of four amino acids (Leu, Tyr, Cys, and Asp); Twenty-one percent of the strains possessed the mutation at codon 516 (GAC to TAC and GTC) that resulted in the conversion of Asp to Tyr and Val respectively, which is in line with previous work [14, 20–22]. Interestingly, a rare mutation at codon 533 (Leu to Pro) was also found in RIF-resistant strains by sequencing analysis, as reported earlier in a few studies [20, 21, 23–25].

New mutations reported in this study include mutation from Arg-Cys at codon 528, Asn-Asp at codon 518, Phe-Leu at codon 506, Leu-Pro at codon 511, Gln-Val/Glu/Pro/Leu at codon 513 and Gln-His at codon 510. Despite a large number of mutations already reported in other studies, the evidence of new mutations in this study indicates that mutations continue to arise, possibly due to the ability of *M. tuberculosis* to adapt drug exposure.

The *rpoB* mutation not detected by the GenoType MDBDR*plus* assay was a deletion at codon 518 of the *rpoB* gene. This region is situated between two probes (WT 4 and 5) and, thus, can be missed due to overlap between these probes. It has been previously reported that deletion at *rpoB* 518 codon missed by another line probe test i.e. the Inno-LiPA Rif.TB. Other Asian countries have also been reported the deletion at 518 [26]. We also found two novel mutations outside the rifampicin-resistant determining region (RRDR) at codon 506 (Phe-Leu) and codon 572 (Ile-Phe). Some novel mutations found inside the RRDR, i.e. 3-nucleotide insertions (Leu-514-515, Pro-511-513and Glu-511-512), which are not reported even globally.

In this study, 28% of the strains possess mutation at codon 513 and 516 in the *rpoB* region. This region of the *rpoB* gene is situated between two probes (WT 3 and 4) and, thus, can be missed due to their overlap. These codons can be added as common mutations in Genotype MTBDR*plus* for detection of rifampicin resistance detection in *Mycobacterium tuberculosis* strains, which increases the sensitivity and specificity of this assay for rifampicin resistance detection. In this study, MUT probes that most commonly failed to hybridize were *rpoB*MUT3 531TTG, due to the absence of WT8 probe and 516GAC and 513CAA mutation due to absence of WT3&4 probes. This may indicate the need for continued assay optimization over the inclusion of novel MUT probes in GenoType MDBDR*plus* assay.

The GenoType MDBDR*plus* assay can observed the mutations in the *rpoB* gene i.e. Ser-531-Leu, His-526-Tyr, His- 526-Asp, and Asp-516-Val, which is the four most frequent mutations in the *rpoB* gene, while this assay indicates only the presence of a genetic alteration for other mutations. Nevertheless, this study and old studies proposed that the frequencies of specific mutations are dissimilar in different countries; hence, additional studies are essential for how to amend the accurate diagnosis of RIF-resistant *M*. *tuberculosis* infections using the GenoType MDBDR*plus* assay.

The limitations of the study are; 1- Phenotypic drug resistance testing (liquid culture or solid culture) was not performed. So the performance of GenoType® MTBDR*plus* was not assessed as compared to the culture method. 2- The lineage of the isolates studied is not known. 3- Sample size of drug resistance isolates are small however it can be considered as a valid preliminary study. 4- Genexpert was not included in the study.

## Conclusions

The absence of wild type probe hybridization without mutation probe hybridization in Genotype MTBDR*plus* assay was primarily the result of the failure of mutation probe hybridization and it results in new or rare mutations using DNA sequencing. Additional probes are necessary to be included in the MTBDR*plus* assay to improve the detection of rifampicin-resistant *Mycobacterium tuberculosis* strains.

## Methods

### Setting

The study was performed at the Department of Microbiology, Institute of Medical Sciences, Banaras Hindu University, Varanasi, India from February 2015 to May 2016. The Mycobacteriology Laboratory is accredited by the National Accreditation Board for Testing and Calibration Laboratories (NABL) for the performance of the MTBDR plus assay and Drug susceptibility test by MGIT and solid method.

### Study design and ethics statement

This study was carried out to find out the diagnostic performance of the GenoType MTBDR*plus* assay in sputum samples in comparison with DNA sequencing methodologies.

The study was ethically approved by the ethical committee of the Institute of Medical Sciences, Banaras Hindu University, Varanasi, India.

### Specimen processing and rifampicin drug resistance

A total of 150 sputum samples obtained from 150 patients were used in this study. The samples were processed using the N-acetyl-L-cysteine-sodium hydroxide (NALC-NaOH) method of digestion and decontamination [27]. After digestion and decontamination, specimens were neutralized and centrifuged at 3000x *g* for 15 min.

### LPA MTBDRplus assay (Hain Lifescience, Nehren, Germany)

The LPA MTBDR*plus* assay was performed on each sample according to the manufacturer’s instructions [28–30]. MTBDR*plus* assay utilizes polymerase chain reaction (PCR) amplification followed by reverse hybridization to specific, immobilized oligonucleotide probes to detect either *Mycobacterium tuberculosis* (MTB) wild type sequences or mutations associated with RIF and INH resistance.

MTBDR*plus* assay was performed on decontaminated sputum sediment. For, DNA extraction, 500 μl of decontaminated sediment was centrifuged and resuspended with 100 μl sterile molecular grade water. Bacterial pellets were subjected to chemical lysis using the Genolyse kit. Multiplex PCR was executed by HotStar *Taq* DNA polymerase 250 U (Qiagen GmBH, Hilden, Germany), for sediment with 40 amplification cycles and for cultured specimens with 30 cycles. The following reaction conditions were used: denaturation at 95 °C for 5 min, followed by 10 cycles of 30 s at 95 °C and 2 min at 58 °C, followed by 20 additional cycles of 25 s at 95 °C, 40 s at 53 °C, and 40 s at 70 °C, ending with a final extension step of 8 min at 70 °C. According to the manufacturer’s instructions hybridization and detection were carried out with a TwinCubator (Hain Lifescience GmbH) semiautomated washing and shaking device and using the reagents provided with the kit.

**Fig. 3:**
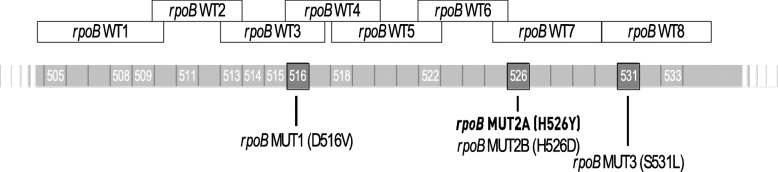
The description of the probes of the MTBDRplus kit on the rpoB sequence in *Mycobacterium tuberculosis*

For the detection of RIF resistance, the MTBDR*plus* assay strip contains 8 *rpoB* wild type probes (including codons 505 to 533); D516V, D526Y, H526D, and S531L, the four mutant probes in the *rpoB* gene that ranges from 505 to 533 codon (Fig. 3).

The interpretation of MTBDR*plus* assay has been done by follow these methods*.* If on the test strip one or more MUT probes hybridize and one or more WT probes fail to hybridize or if one or more WT probes fail to hybridize (without MUT probe hybridization) then that *MTB* strains are resistant. The absence of one or more WT probes hybridization and failure of the MUT probe hybridization provide an indirect method to identify novel or rare mutations located in those regions covered by the specific WT probes [31].

### DNA sequencing

#### Primer design

Novel PCR primer was designed for the amplification of the *rpoB* gene. Primer pairs for amplification of the *rpoB* gene were intended using the genome sequence of *M. tuberculosis* H37Rv strain (GenBank accession no. NC_000962) as reference. Melting temperature, secondary structure formation, and potential primer-dimer formation were find out using LaserGene 9.1 (DNAStar, Madison, WI) and Primer Express 3.0 (Life Technologies, Foster City, CA).

### Sequencing of *rpoB* gene

**Fig. 4:**
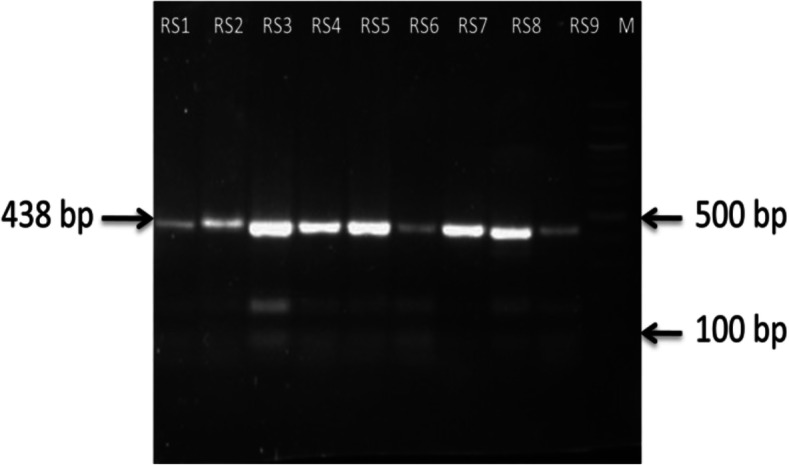
Electrophoresis gel of PCR results for sequencing of *rpoB* gene in *Mycobacterium tuberculosis*

DNA sequencing was performed on all clinical specimens found to be non-interpretable on LPA MTBDR*plus* assay by Sanger sequencing. The regions of *rpoB* associated with Rif^r^ were sequenced after amplification by PCR. The forward RSF (5′-GATGACCACCCAGGACGTGGAG-3′) and reverse RSR (5′-TCGATCGGCGAATTGGCCTGTG-3′) primers used for both PCR and sequencing to amplify a 438-bp fragment of the *rpoB* gene containing the 81-bp hypervariable region (Fig. 4). The 25 μl PCR reactions were performed with 5 μl 10X Q5 reaction buffer (New England BioLabs), 1 μl 200 μM of each dNTP, Q5 hot start *Taq* DNA polymerase (New England BioLabs), 5 μl Q5 GC enhancer (New England BioLabs), 10 μl of DNA from the specimen and 10 pmol of each primer (GeNei, Banglore, India) and added Milli Q to create a total volume of 25 μl.

### Reaction condition

Each reaction was initiated at 98 °C for 30 s, run 35 cycles of (98 °C for 10s, 66 °C for 25 s, 72 °C for 30s) and finally elongated at 72 °C for 2 min.

### Visualization of PCR product

The PCR product was confirmed on an agarose gel to be approximately 438 bp.

### Sequencing and assembly

All amplicons were subjected to DNA sequencing (Sci Genome, Cochin, Kerala, India). The obtained sequences were assembled manually, and polymorphisms detection was achieved by comparison with the available sequences for the *rpoB* gene of *M. tuberculosis* H37Rv sequence. The new mutations were analyzed using clustal Omega programs. The genome sequencing has been deposited at DDBJ/EMBL/GenBank. These are under the processing (Supplimetary material-1).

## Supplementary information

**Additional file 1**

## Data Availability

The datasets used and/or analyzed during the current study are available from the corresponding author on reasonable request.

## Abbreviations

MTBC: *Mycobacterium tuberculosis* complex
WT: Wild type
HIV: Human immunodeficiency virus
LPA: Line Probe Assay
INH: Isoniazid
RIF: Rifampicin
MDR-TB: Multidrug resistant-tuberculosis
PCR: Polymerase chain reaction
RRDR: Rifampicin-resistant determining region
