# Supplementary material for: Detection of mutations in the rpoB gene of rifampicin-resistant Mycobacterium tuberculosis strains inhibiting wild type probe hybridization in the MTBDR plus assay by DNA sequencing directly from clinical specimens
Source: BMC Microbiol. 2020 Sep 16;20:284. doi: 10.1186/s12866-020-01967-5 (PMC7493411; doi:10.1186/s12866-020-01967-5)
Supplement: Supplementary file 1 — Additional file 1 [file 12866_2020_1967_MOESM1_ESM.docx]

CLUSTAL O(1.2.4) multiple sequence alignment

Seq17[organism= MTTQDVEAITPQTLINIRPVVAAIKEFFGTSQLRSQFMDQNNPLSGLTHKRRLSALGPGG 60

Seq21 MTTQDVEAITPQTLINIRPVVAAIKEFFGTSQPLSQFMDQNNPLSGLTHKRRLSALGPGG 60

Seq13[organism= MTTQDVEAITPQTLINIRPVVAAIKEFFGTSQL-SQFMDQ-NPLSGLTHKRRLSALGPGG 58

Seq18[organism= MTSQDVEAITPQTLINIRPVVAAIKEFFGTSQL-SQFMDQNNPLSGLTHKRRLSALGPGG 59

Seq22[organism= MTTQDVEAITPQTLINIRPVVAAIKEFFGTSQL-SEFMDQNNPLSGLTHKRRLSALGPGG 59

Seq19[organism= MTTQDVEAITPQTLINIRPVVAAIKELFGTSQL-SQFMDQNNPLSGLTHKRRLSALGPGG 59

Seq16[organism= MTTQDVEAITPQTLINIRPVVAAIKEFFGTSQP-SQFMDQNNPLSGLTHKRRLSALGPGG 59

Seq20[organism= MTTQDVEAITPQTLINIRPVVAAIKEFFGTSQP-SQFMDQNNPLSGLTHKRRLSALGPGG 59

Seq15[organism= MTTQDVEAITPQTLINIRPVVAAIKEFFGTSQL-SQFLDQNNPLSGLTHKRRLSALGPGG 59

Seq14[organism= MTTQDVEAITPQTLINIRPVVAAIKEFFGTSQL-SQFMDQDNPLSGLTHKRRLSALGPGG 59

Seq12[organism= MTTQDVEAITPQTLINIRPVVAAIKEFFGTSQL-SQFMDQNNPLSGLTCKRRLSALGPGG 59

Seq11[organism= MTTQDVEAITPQTLINIRPVVAAIKEFFGTSQL-SQFMDQNNPLSGLTHKCRLSALGPGG 59

Seq10[organism= MTTQDVEAITPQTLINIRPVVAAIKEFFGTSQL-SQFMDQNNPLSGLTNKRRLSALGPGG 59

Seq9[organism= MTTQDVEAITPQTLINIRPVVAAIKEFFGTSQL-SQFMDQNNPLSGLTLKRRLSALGPGG 59

Seq8[organism= MTTQDVEAITPQTLINIRPVVAAIKEFFGTSQL-SQFMYQNNPLSGLTHKRRLSALGPGG 59

Seq7[organism= MTTQDVEAITPQTLINIRPVVAAIKEFFGTIQL-SQFMDQNNPLSGLTHKRRLSALGPGG 59

Seq6[organism= MTTQDVEAITPQTLINIRPVVAAIKEFFGTSQL-SQFMVQNNPLSGLTHKRRLSALGPGG 59

SeqN MTTQDVEAITPQTLINIRPVVAAIKEFFGTSQL-SQFMDQNNPLSGLTHKRRLSALGPGG 59

Seq1 MTTQDVEAITPQTLINIRPVVAAIKEFFGTSQL-SQFMDQNNPLSGLTHKRRLWALGPGG 59

Seq2[organism= MTTQDVEAITPQTLINIRPVVAAIKEFFGTSQL-SQFMDQNNPLSGLTHKRRLFALGPGG 59

Seq5[organism= MTTQDVEAITPQTLINIRPVVAAIKEFFGTSQL-SQFMDQNNPLSGLTHKRRLFALGPGG 59

Seq3[organism= MTTQDVEAITPQTLINIRPVVAAIKEFFGTSQL-SQFMDQNNPLSGLTHKRRLYALGPGG 59

Seq4[organism= MTTQDVEAITPQTLINIRPVVAAIKEFFGTSQL-SQFMDQNNPLSGLTHKRRLCALGPGG 59

**:***********************:*** * *:*: * ******* * ** ******

Seq17[organism= LSRERAGLEVRDVHPSHYGRMCPIETPEGPNIGLIGSLSVYARVNPFGFIETPYRKVVDG 120

Seq21 LSRERAGLEVRDVHPSHYGRMCPIETPEGPNIGLIGSLSVYARVNPFGFIETPYRKVVDG 120

Seq13[organism= LSRERAGLEVRDVHPSHYGRMCPIETPEGPNIGLIGSLSVYARVNPFGFIETPYRKVVDG 118

Seq18[organism= LSRERAGLEVRDVHPSHYGRMCPIETPEGPNIGLFGSLSVYARVNPFGFIETPYRKVVDG 119

Seq22[organism= LSRERAGLEVRDVHPSHYGRMCPIETPEGPNIGLIGSLSVYARVNPFGFIETPYRKVVDG 119

Seq19[organism= LSRERAGLEVRDVHPSHYGRMCPIETPEGPNIGLIGSLSVYARVNPFGFIETPYRKVVDG 119

Seq16[organism= LSRERAGLEVRDVHPSHYGRMCPIETPEGPNIGLIGSLSVYARVNPFGFIETPYRKVVDG 119

Seq20[organism= LSRERAGLEVRDVHPSHYGRMCPIETPEGPNIGLIGSLSVYARVNPFGFIETPYRKVVDG 119

Seq15[organism= LSRERAGLEVRDVHPSHYGRMCPIETPEGPNIGLIGSLSVYARVNPFGFIETPYRKVVDG 119

Seq14[organism= LSRERAGLEVRDVHPSHYGRMCPIETPEGPNIGLIGSLSVYARVNPFGFIETPYRKVVDG 119

Seq12[organism= LSRERAGLEVRDVHPSHYGRMCPIETPEGPNIGLIGSLSVYARVNPFGFIETPYRKVVDG 119

Seq11[organism= LSRERAGLEVRDVHPSHYGRMCPIETPEGPNIGLIGSLSVYARVNPFGFIETPYRKVVDG 119

Seq10[organism= LSRERAGLEVRDVHPSHYGRMCPIETPEGPNIGLIGSLSVYARVNPFGFIETPYRKVVDG 119

Seq9[organism= LSRERAGLEVRDVHPSHYGRMCPIETPEGPNIGLIGSLSVYARVNPFGFIETPYRKVVDG 119

Seq8[organism= LSRERAGLEVRDVHPSHYGRMCPIETPEGPNIGLIGSLSVYARVNPFGFIETPYRKVVDG 119

Seq7[organism= LSRERAGLEVRDVHPSHYGRMCPIETPEGPNIGLIGSLSVYARVNPFGFIETPYRKVVDG 119

Seq6[organism= LSRERAGLEVRDVHPSHYGRMCPIETPEGPNIGLIGSLSVYARVNPFGFIETPYRKVVDG 119

SeqN LSRERAGLEVRDVHPSHYGRMCPIETPEGPNIGLIGSLSVYARVNPFGFIETPYRKVVDG 119

Seq1 LSRERAGLEVRDVHPSHYGRMCPIETPEGPNIGLIGSLSVYARVNPFGFIETPYRKVVDG 119

Seq2[organism= LSRERAGLEVRDVHPSHYGRMCPIETPEGPNIGLIGSLSVYARVNPFGFIETPYRKVVDG 119

Seq5[organism= LSRERAGLEVRDVHPSHYGRMCPIETPEGPNIGLIGSLSVYARVNPFGFIETPYRKVVDG 119

Seq3[organism= LSRERAGLEVRDVHPSHYGRMCPIETPEGPNIGLIGSLSVYARVNPFGFIETPYRKVVDG 119

Seq4[organism= LSRERAGLEVRDVHPSHYGRMCPIETPEGPNIGLIGSLSVYARVNPFGFIETPYRKVVDG 119

**********************************:*************************

Seq17[organism= VVSDEIVYLTADEEDRHVVAQANSPIX 147

Seq21 VVSDEIVYLTADEEDRHVVAQANSPIX 147

Seq13[organism= VVSDEIVYLTADEEDRHVVAQANSPIX 145

Seq18[organism= VVSDEIVYLTADEEDRHVVAQANSPIX 146

Seq22[organism= VVSDEIVYLTADEEDRHVVAQANSPIX 146

Seq19[organism= VVSDEIVYLTADEEDRHVVAQANSPIX 146

Seq16[organism= VVSDEIVYLTADEEDRHVVAQANSPIX 146

Seq20[organism= VVSDEIVYLTADEEDRHVVAQANSPIX 146

Seq15[organism= VVSDEIVYLTADEEDRHVVAQANSPIX 146

Seq14[organism= VVSDEIVYLTADEEDRHVVAQANSPIX 146

Seq12[organism= VVSDEIVYLTADEEDRHVVAQANSPIX 146

Seq11[organism= VVSDEIVYLTADEEDRHVVAQANSPIX 146

Seq10[organism= VVSDEIVYLTADEEDRHVVAQANSPIX 146

Seq9[organism= VVSDEIVYLTADEEDRHVVAQANSPIX 146

Seq8[organism= VVSDEIVYLTADEEDRHVVAQANSPIX 146

Seq7[organism= VVSDEIVYLTADEEDRHVVAQANSPIX 146

Seq6[organism= VVSDEIVYLTADEEDRHVVAQANSPIX 146

SeqN VVSDEIVYLTADEEDRHVVAQANSPIX 146

Seq1 VVSDEIVYLTADEEDRHVVAQANSPIX 146

Seq2[organism= VVSDEIVYLTADEEDRHVVAQANSPIX 146

Seq5[organism= VVSDEIVYLTADEEDRHVVAQANSPIX 146

Seq3[organism= VVSDEIVYLTADEEDRHVVAQANSPIX 146

Seq4[organism= VVSDEIVYLTADEEDRHVVAQANSPIX 146

***************************
